# A *de novo* reference transcriptome for *Bolitoglossa vallecula*, an Andean mountain salamander in Colombia

**DOI:** 10.1016/j.dib.2020.105256

**Published:** 2020-02-11

**Authors:** Claudia M. Arenas Gómez, M. Ryan Woodcock, Jeramiah J. Smith, S. Randal Voss, Jean Paul Delgado

**Affiliations:** a Universidad de Antioquia. Sede de Investigación Universitaria. Torre 2, laboratorio 432. Calle 62 No. 52 – 59. Medellín, Colombia; b Current address: Marine Biological Laboratory, Eugene Bell Center for Regenerative Biology and Tissue Engineering, Woods Hole, 02543 MA, USA; c Department of Science, Math, and Technology, Medaille College, Buffalo, NY, 14214, USA; d Department of Biology, University of Kentucky. Lexington, KY 40506, USA; e Department of Neuroscience, Spinal Cord and Brain Injury Research Center, University of Kentucky. Lexington, KY 40536, USA

**Keywords:** *Bolitoglossa*, Plethodontid, Salamanders, Skin, Transcriptomics

## Abstract

The amphibian order Caudata, contains several important model species for biological research. However, there is need to generate transcriptome data from representative species of the primary salamander families. Here we describe a *de novo* reference transcriptome for a terrestrial salamander, *Bolitoglossa vallecula* (Caudata: Plethodontidae). We employed paired-end (PE) illumina RNA sequencing to assemble a *de novo* reference transcriptome for *B. vallecula*. Assembled transcripts were compared against sequences from other vertebrate taxa to identify orthologous genes, and compared to the transcriptome of a close plethodontid relative (*Bolitoglossa ramosi*) to identify commonly expressed genes in the skin. This dataset should be useful to future comparative studies aimed at understanding important biological process, such as immunity, wound healing, and the production of antimicrobial compounds.

**Table:** Specifications Table

Subject	Animal Science and Zoology
Specific subject area	Wild caught adult salamanders of the specie *Bolitoglossa vallecula* from the Andes region of Antioquia, Colombia.
Type of data	RNA Sequencing Data
How data were acquired	Paired-End sequenced (2x 100 bp) using an Illumina Hiseq-2000
Data format	Raw Sequencing reads, assembled contigs and preliminary annotation.
Parameters for data collection	Adult animals were used to surgically collect multiple tissues (limb, skin, heart).
Description of data collection	Tissues were collected from animals following euthanasia via immersion in 2% of MS-222 followed by decapitation. All samples (limb, skin, heart) were stored at −20 °C in Trizol® reagent by one week until total RNA was extracted individually from each tissue. Paired-End sequenced (2x 100 bp) using an Illumina Hiseq-2000.
Data source location	Institution: Universidad de Antioquia City/Town/Region: Antioquia Country: Colombia Latitude and longitude (and GPS coordinates) for collected samples/data: 6°18'16.0"N 75°08'06.0"W
Data accessibility	Repository name: Sequence Read Archive Data identification number: SRP125550 Direct URL to data: https://www.ncbi.nlm.nih.gov/sra?term=SRP125550 Repository name: The Gene Expression Omnibus (GEO) Data identification number: GSE105232 Direct URL to data: https://www.ncbi.nlm.nih.gov/geo/query/acc.cgi?acc=GSE107213 Repository name: Transcriptome Shotgun Assembly DDBJ/EMBL/GenBank Data identification number: GHME01000000 Direct URL to data: https://www.ncbi.nlm.nih.gov/Traces/wgs/GHME01 The output of transcriptome annotation using various strategies is included in additional tables (Supplementary tables 1-8).

**Table:** 

**Value of the Data** • We describe a *de novo* reference transcriptome for a terrestrial salamander, *Bolitoglossa vallecula* (Caudata: Plethodontidae). • Few transcriptomic data exist for plethodontids, further sampling of genes sequences across additional caudate families is needed in order to better understand how evolution has maintained and diversified pathways that contribute to key biological processes, such as: development, tissue regeneration, antipredator defenses, and the establishment/maintenance of microbial interactions. • This dataset should be useful to future comparative studies aimed at understanding important biological process, including immunity, wound healing, and the production of antimicrobial compounds.

## 1 Data description

### 1.1 *De novo* transcriptome assembly

**Fig. 1:**
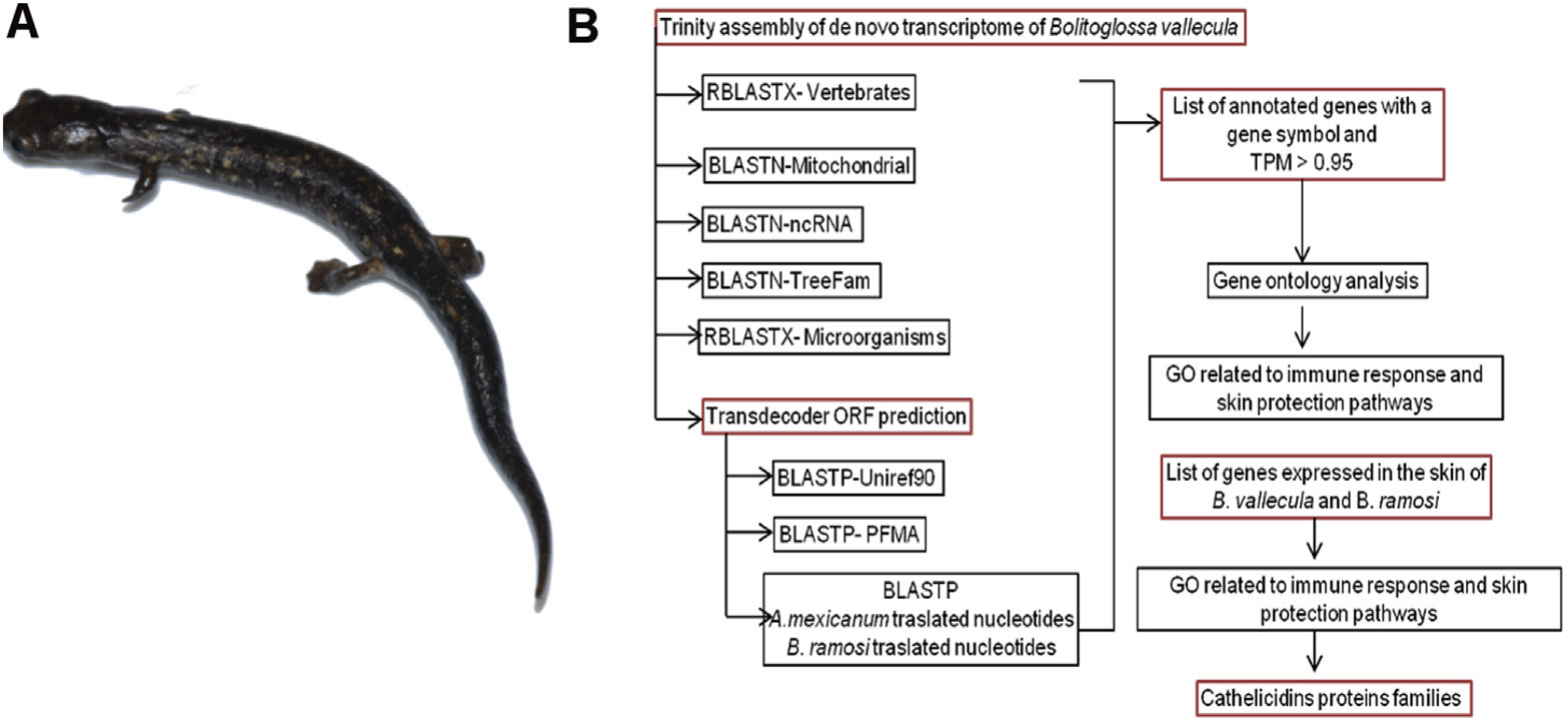
De novo reference transcriptome of *Bolitoglossa vallecula*. A. *Bolitoglossa vallecula* is a salamander that belongs to the family Plethodontidae and can be found in interior forests ranging in elevation from 1200 to 3000 m above sea level in the Andean mountains of Colombia. B. Workflow used to perform annotation of the *de novo* reference transcriptome of *B. vallecula* and identification of proteins important for skin protection.

In this dataset, we present a *de novo* reference transcriptome of *Bolitoglossa vallecula* (Caudata: Plethodontidae) (Fig. 1A and B), a terrestrial salamander from the Andes. The genome size of *B. vallecula* was estimated to be ∼25 Gb using flow cytometry of propidium iodide-stained nuclei. The depth of sequencing for each sample was approximately 50 million reads (Table 1).

**Table 1:** Transcript sequence depth for samples used in this dataset.

Sample	# of sequence reads
Limb_1	54,273,832
Limb_2	47,043,730
Skin	53,957,582
Heart	50,546,302

A reference transcriptome was assembled to recover transcripts and isoforms from all samples with a minimal length of 200 nucleotides. The total number of high quality assembled PE reads recovered was 198,261,418. Using the Trinity assembler, we obtained 257,727 contigs with a GC content of 44.15% and an average length of 912 bp, with a maximum assembled contig length of 20,962 bp (Table 2).

**Table 2:** Trinity assembly summary statistics of the *de novo* reference transcriptome for a non-model terrestrial salamander, *Bolitoglossa vallecula* (Caudata: Plethodontidae).

PARAMETER	NUMBER
Total aligned reads	200,938,561
Total number of high quality assembled paired-end reads	198,261,418
Total trinity transcripts	257,727
Total trinity 'genes'	204,067
Average 'genes' length (pb)	912
Total GC count (%)	44,15
N50 E90N50	2065 3453
Longest contig (bp)	20,962
Shortest contig	200
Number of contigs > 200 bp	201,731
Number of contigs > 1 Kb	49,646
Number of contigs > 5 Kb	6073
Number of contigs > 10 Kb	277
Number of predict ORF (transdecoder)	85,762

### 1.2 Gene annotation

Assembled transcripts were assigned to gene families using translated BLAST (blastx) searches against the TreeFam database [1]. Transcript annotation for non-coding RNAs (ncRNAs) was accomplished by nucleotide BLAST against sequences downloaded from the miRBase [2] and RFam [3] databases. We further predicted long open reading frames (ORF) using TransDecoder software (version 3.0.0) [4] and searched for additional homologs using protein BLAST against the UniRef90 [5] and PFAM database [6]. Possible contaminants in the transcriptome were filtered using reciprocal best hits of translated BLAST searches (RBH-Blast) to sequences of bacteria, viruses, single-celled eukaryotes, fungi, and ribosomal and mitochondrial sequences of salamander's batch downloaded from NCBI database (ftp://ftp.ncbi.nlm.nih.gov/genomes/). Putative orthologs were identified through RBH-Blast for seven vertebrate taxa available through the Ensembl database or RefSeq NCBI (Table 3, Supplementary table 1), namely *Anolis carolinensis* (GCA_000090745.1), *Danio rerio* (GCA_000002035.3), *Gallus gallus* (PRJNA10808), *Homo sapiens* (PRJNA168), *Mus musculus* (GCA_000001635.7), *Xenopus tropicalis* (PRJNA205740), *Latimeria chalumnae* (GCA_000225785.1)*.* Finally, we used TransDecoder predict open reading frames (ORFs) from nucleotide databases of three salamander species: *A. mexicanum* [7], *Notophtalmus viridescen*s [8] and *B. ramosi* [9], then performed protein BLAST searches against predicted ORFs from our *B. vallecula* assembly in order to identify homologous sequences in other salamanders. These were subsequently filtered to retain candidate orthologs with amino acid identity exceeding 70%. Annotated genes (TPM ≥ 0.95) were queried to identify Gene Ontology (GO) categories and signaling pathways using PANTHER Data Base (Version 11.1) [10].

**Table 3:** Summary of homology searches based on alignment of the *B. vallecula* reference transcriptome to several vertebrate species.

Vertebrates	Orthologs
Ambystoma mexicanum	16951
Anolis carolinensis	8033
Bolitoglossa ramosi	28528
Danio rerio	7143
Gallus gallus	7472
Homo sapiens	6779
Latimeria chalumnae	8164
Mus musculus	8245
Notophalmus viridescens	16959
Xenopus tropicalis	8296

We identified presumptive homologs for 13% (n = 33,400) of *B. vallecula* reference transcripts (Table 3), including 6779 transcripts (non-redundant) that were orthologous to a known human gene. Additionally, translated ORFs from *B. vallecula* were queried (protein BLAST) against caudate sequence data, including the translated nucleotide databases for *Ambystoma mexicanum*, *Notophalmus viridescens* and *Bolitoglossa ramosi* (Table 3, Supplementary table 2). The homology sequences (by percent of identity) between the *Bolitoglossa* sp. were higher than other salamanders.

**Fig. 2:**
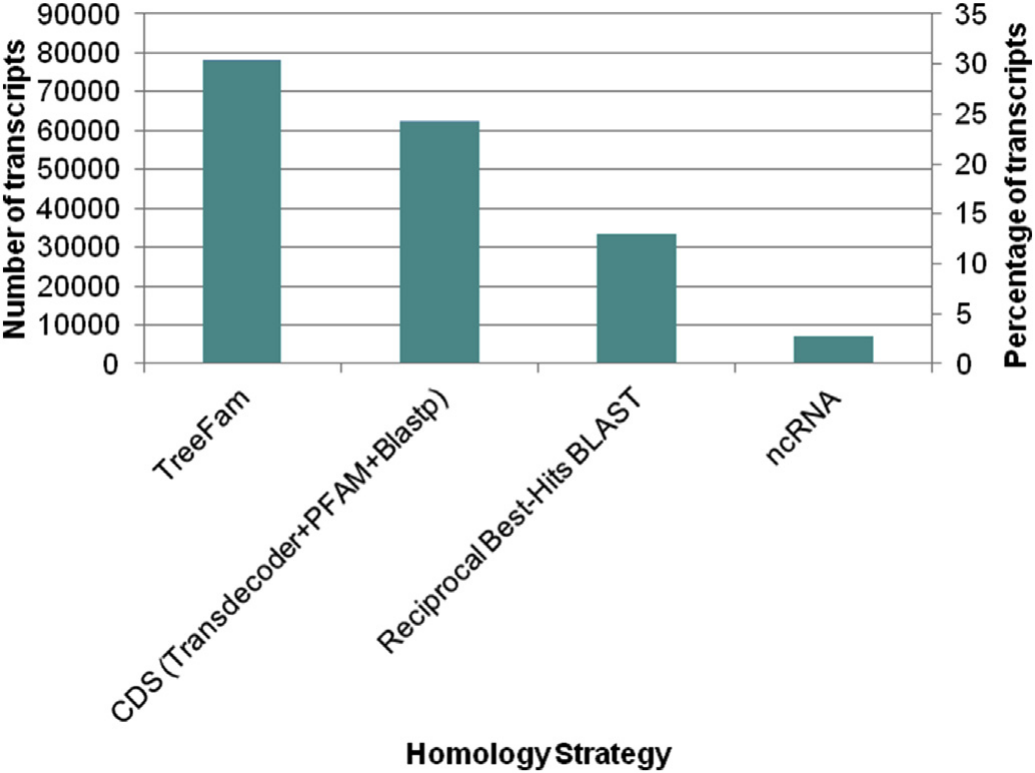
Homology assignments recovered *for B. vallecula* transcripts. The *B. vallecula* transcriptome was surveyed by Reciprocal Best Hits of translation BLAST searches (RBH-Blast) to predicted protein or translated databases from seven vertebrate taxa; namely *Anolis carolinensis*, *Danio rerio*, *Gallus gallus*, *Homo sapiens*, *Mus musculus*, *Xenopus tropicalis*, *Latimeria chalumnae*. Additional gene family homologs were assigned to *B. vallecula* using protein BLAST against the UniRef90 (Suzek et al., 2014) and PFAM domains (Finn et al. 2016) databases.

Transcripts that did not return a significant sequence alignment in the BLAST searches described above (30.3%, 78,077) were queried against the TreeFam database (Fig. 2, Supplementary table 3). Transcripts without a gene family match in TreeFam (2.8%, n = 7111), were further queried against the (miRBas and RFam) ncRNA databases (Fig. 2, Supplementary table 4).

Additionally, complete open reading frames (ORFs) were predicted by TransDecoder software for 33% (n = 85,762) of the unannotated transcripts. From these ORF translations, there were 49,721 and 59,325 transcripts recovered from queries of UniRef90 and PFAM, respectively (Supplementary table 5). In total, using this strategy we recovered information for 62,274 (24%) non-redundant transcripts. We also identified 18 presumptive mitochondrial transcripts for *B. vallecula* (Supplementary table 6). Finally, translated nucleotide BLAST (tblastn) searches were also performed against microorganism sequences to identify potential contaminants (possible microbiote components) of the *B. vallecula* transcriptome, 0.73% (n = 1901) of the transcriptome was likely exogenous to *B. vallecula* (Supplementary table 6) and of these, 582 transcripts were also present in the *B. ramosi* transcriptome.

### 1.3 GO analysis

**Fig. 3:**
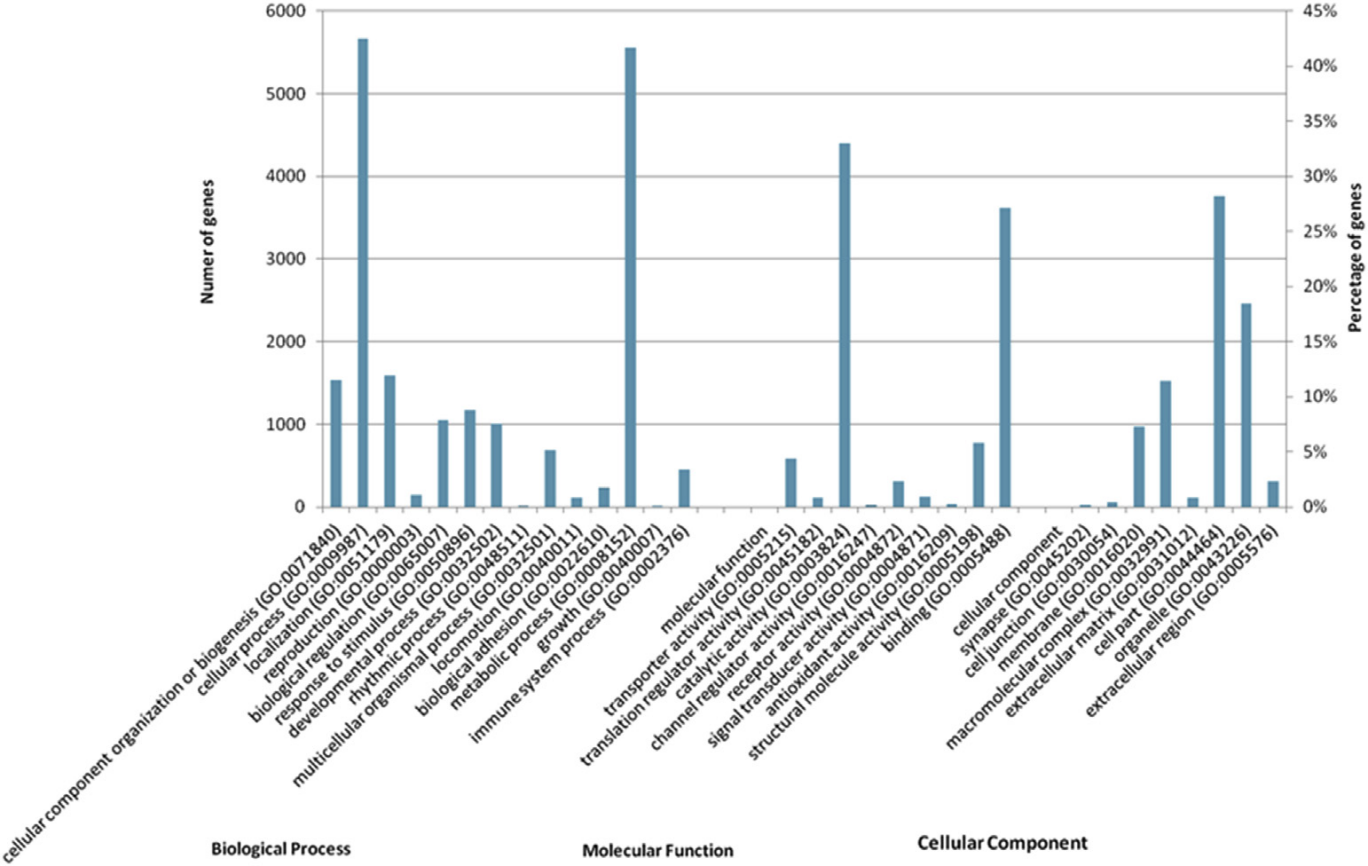
Distribution of Gene Ontology (GO) categories for sampled *Bolitoglossa vallecula* transcripts. Gene ontology Level 2 categories for Biological process, Molecular function and Cellular component.

**Fig. 4:**
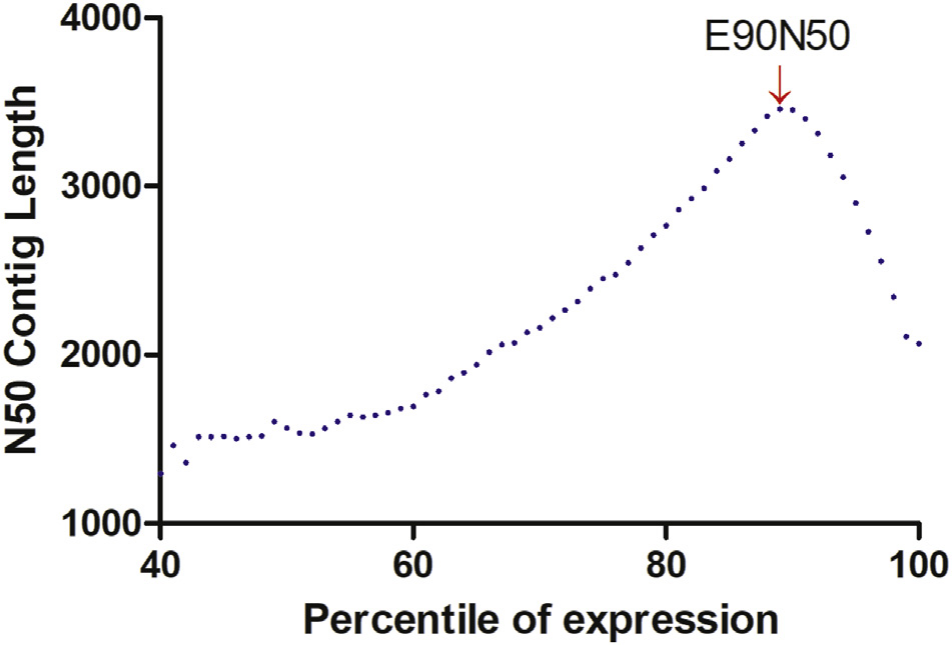
Expression-based contiguity statistics for the *B. vallecula* de *novo* reference transcriptome. The assembly has E90N50 of >3kb (red arrow).

Gene ontology analyses were conducted using 6641 non-redundant transcripts with defined human orthologs (Fig. 3, Supplementary table 7). The Panther Database clustered these genes into broad “cellular” (GO:0009987) and “metabolic process” (GO:0008152) GO terms (Fig. 4). Integrin signaling (P00034) and CCKR signaling (P06959) (Table 4), were among the most enriched pathways in *B. vallecula* skin transcriptome. Pathways associated with innate immunity and injury response include genes in the PI3 kinase pathway including: *C1QB*, *CDK1* and *MAPKAPK3*; some of which have also been previously described in the proteome of salamander skin [11]. Other genes related to injury response include: *SMAD1*, *TNC*, and *PTK7* and genes that contribute to EGF receptor signaling (P00018: *TGFA*, *STAT5A*, *RAC2*), p38 MAPK (P05918: *GADD45A*, *SRF*, *MAP3K7*) and Ras (P04393: *RAC2*, *RAC3*, *TIAM1*) pathways.

**Table 4:** Assignment of Bolitoglossa vallecula genes to signaling pathways reported in the Panther pathways database.

Pathway (Panther code)	Number of Genes	(P-value)
Ubiquitin proteasome pathway (P00060)	51	3.17E-06
General transcription regulation (P00023)	27	1.51E-02
Transcription regulation by bZIP transcription factor (P00055)	37	7.10E-03
Parkinson disease (P00049)	56	9.85E-03
CCKR signaling map (P06959)	92	4.62E-04
Integrin signalling pathway (P00034)	90	4.57E-02
Unclassified (UNCLASSIFIED)	5594	0.00E+00

### 1.4 Homology comparisons between *Bolitoglossa* sp

In a previous study [9], we assembled a reference transcriptome for *B. ramosi* that included transcripts derived from skin tissue. We compared the *B. vallecula* and *B. ramosi* skin datasets to identify commonly expressed transcripts between the two species. We recovered 4007 orthologous genes that were expressed in the skin transcriptome of both *B. ramosi* and *B. vallecula* (Supplementary table 8). GO terms associated with immune system responses, including immunomodulation and skin barrier integrity were identified within this common set of skin transcripts (Table 5). This shared skin transcriptome also included genes associated with response to stimulus (GO: 0050896), such as *TXLNA* and *TXLNG* (antibacterial response proteins).

**Table 5:** Top 20 most representative immune response gene ontologies identified using homologous genes identified from the skin of *Bolitoglossa vallecula* and *Bolitoglosa ramosi*.

Gene ontology process associated to Immune response	Number of genes
activation of immune response (GO:0002253)	101
immune response-activating signal transduction (GO:0002757)	96
immune response-regulating cell surface receptor signaling pathway (GO:0002768)	86
regulation of innate immune response (GO:0045088)	78
negative regulation of immune system process (GO:0002683)	65
positive regulation of innate immune response (GO:0045089)	62
immune response-activating cell surface receptor signaling pathway (GO:0002429)	59
innate immune response-activating signal transduction (GO:0002758)	55
activation of innate immune response (GO:0002218)	55
regulation of immune effector process (GO:0002697)	49
immune response-regulating cell surface receptor signaling pathway involved in phagocytosis (GO:0002433)	32
negative regulation of immune response (GO:0050777)	20
adaptive immune response (GO:0002250)	18
positive regulation of immune effector process (GO:0002699)	18
regulation of leukocyte mediated immunity (GO:0002703)	18
humoral immune response (GO:0006959)	18
leukocyte activation involved in immune response (GO:0002366)	17
cell activation involved in immune response (GO:0002263)	17
regulation of adaptive immune response (GO:0002819)	17
leukocyte mediated immunity (GO:0002443)	14

## 2 Experimental design, materials, and methods

### 2.1 Animals and surgical procedures

All animals used in this work were collected under the Contract on Genetic Access for scientific research for non-commercial profit (Contrato de acceso a recursos genéticos para la investigación científica sin interés commercial) resources number 118–2015, which was provided by the Ministerio del Medio Ambiente (Ministry of Environment) of Colombia to the Principal Investigator. The Institutional Bioethics and Animal Care and Use Committee of the University of Antioquia (Medellín, Colombia) approved all experimental procedures. Wild caught adult salamanders (7–10 cm snout to tail length) of the species *Bolitoglossa vallecula* were collected by the night-time visual encounter method [12] in the Andes region of Antioquia, Colombia. Specimens were kept in the laboratory under established protocols for environmental conditions and maintenance [13].

Adult animals (n = 4) were used to surgically collect multiple tissues (limb, skin, heart). Tissues were collected from animals following euthanasia via immersion in 2% of MS-222 followed by decapitation. All samples (limb, skin, heart) were stored at −20 °C in Trizol® reagent for one week until total RNA was extracted individually from each tissue using the manufacturer recommended protocol (Life Technologies).

### 2.2 Illumina sequencing

The quality of RNA samples was assessed by Macrogen using an Agilent 2100 Bioanalyzer. Only samples with RNA integrity number (RIN) of eight or greater were used for further procedures. Sequencing libraries were prepared using the Truseq RNA kit and the resulting library was paired-end (PE) sequenced (2x 100 bp) using an Illumina Hiseq-2000.

### 2.3 Transcript abundance (RSEM)

We used the RSEM (RNA-Seq by Expectation Maximization) alignment-based method to obtain estimates of transcript abundance [14]. Using the RSEM software package, sequence reads were aligned to the reconstructed transcriptome with Bowtie2 [15] and alignments were processed to estimate relative levels of transcription (Transcripts Per Million, TPM).

### 2.4 Data records

The raw sequence reads have been deposited in the Sequence Read Archive under the accession number SRP120553. A total of four different animals were used to obtain limb tissues (n = 2 animals in one pool), heart (n = 1 animal) and skin (n = 1 animal). Transcriptional estimates generated by RSEM are deposited in the Gene Expression Omnibus (GEO) under the accession number GSE105232. The Transcriptome Shotgun Assembly project has been deposited at DDBJ/EMBL/GenBank under the accession GHME00000000. The version described in this paper is the first version, GHME01000000. The output of various annotation strategies is included in supplementary tables (Reciprocal Best Hits of translation BLAST searches to predicted protein or translated databases, protein BLAST against the UniRef90 and PFAM domains databases, orthologous genes inferred by TreeFam, nucleotide BLAST against ncRNA databases, protein BLAST of predicted ORFs to translated nucleotide databases of salamanders).

### 2.5 Genome size (C-value) calculation for *B. vallecula*

The genome size of *B. vallecula* was tested to confirm the DNA contained within one copy of a single genome. The protocol of Hare and Johnston (2011) [16] was follow. Red blood cells (5–10 μl) were isolated from amputated limbs (N = 3) used for flow cytometry. Samples were suspended in EDTA (pH 7.4, 0.126 mM) and fixed in methanol overnight. Thereafter, the samples were incubated in a solution of RNase (10 mg/ml), Triton X-100 (0.1% v/v), EDTA (0.126 mM) and stained with Propidium iodide (0.1 mg/ml) for 30 minutes. The fluorescence intensity was measured in a BDFACSCanto™ II flow cytometer. Chicken Red blood cells (DNA QC particles kit, USA) were used as a control. The genome size was calculated by comparison with the reference control (*Gallus gallus*) using the calculation of Hare and Johnston (2011):

$$GSunk=GSref*\frac{PI_{fluorUnk}}{PI_{fluorRef}}$$

where, *GSunk* = genome size unknown, GSref = genome size of reference, *PI-fluorUnk* = the fluorescent intensity of propidium iodide of unknown sample and *PI-fluorRef* = the fluorescent intensity of propidium iodide of reference sample.

## CRediT author statement

**Claudia M. Arenas Gómez:** Investigation, Methodology, Data curation, Writing - original draft, Formal analysis. **M.Ryan Woodcock:** Methodology, Data curation, Writing - review & editing. **Jeramiah J. Smith:** Methodology, Writing - review & editing. **S. Randal Voss:** Methodology, Writing - review & editing. **Jean Paul Delgado:** Funding acquisition, Writing - review & editing.

## Appendix A Supplementary data

Multimedia component 1 Multimedia component 1

Supp table 1 Output of RBH-blast comparing the *B. vallecula* reference transcriptome with seven different vertebrate databases, including homology assignment (ortholog, paralog). Supp table 1

Supp table 2 Output of the protein BLAST of predicted ORFs from the *B. vallecula* reference transcriptome aligned to translated nucleotide databases of three salamanders (*A.mexicanun*, *N.viridescens* and *B. ramosi*). All hits with >70% amino acid identity > 70 are listed. Supp table 2

Supp table 3 Orthologous genes inferred by TreeFam database present in the *B. vallecula* reference transcriptome. Supp table 3

Supp table 4 Output from the nucleotide BLAST of the *B. vallecula* reference transcriptome against ncRNA databases (miRBas and RFam). Supp table 4

Supp table 5 Output from the protein BLAST of the TransDecoder ORF predictions PFAM and UniRef90 databases. Supp table 5

Supp table 6 Summary table of the RBH-blast hits (%identity) of the sequences from the *B. vallecula* reference transcriptome against different vertebrate and microorganism databases. The hits were assigned to specific categories, including: bacterial contaminants, biota conserved, eukaryote conserved, eukaryote contaminant, fungal contaminant, mitoribosomal contaminant, ophisthokonta conserved, sacropterygii conserved, teleost conserved, urodela conserved, lineage specific, vertebrate conserved, zoonotic virus. Supp table 6

Supp table 7 Transcripts that have TPM support ≥ 0.95 and homology with named human genes. Supp table 7

Supp table 8 Transcripts that have homology with *B. ramosi*, TPM support ≥ 0.95 in skin tissue, and homology with named human genes. Supp table 8
